# Too Late! Influence of Temporal Delay on the Neural Processing of One’s Own Incidental and Intentional Action-Induced Sounds

**DOI:** 10.3389/fnins.2020.573970

**Published:** 2020-11-05

**Authors:** Nina Heins, Ima Trempler, Karen Zentgraf, Markus Raab, Ricarda I. Schubotz

**Affiliations:** ^1^Department of Psychology, University of Münster, Münster, Germany; ^2^Otto Creutzfeldt Center for Cognitive and Behavioral Neuroscience, University of Münster, Münster, Germany; ^3^Institute for Sport Sciences, Goethe University Frankfurt, Frankfurt, Germany; ^4^Institute of Psychology, German Sport University Cologne, Cologne, Germany; ^5^School of Applied Sciences, London South Bank University, London, United Kingdom

**Keywords:** action sounds, action-effect association, delayed auditory feedback, supplementary motor area, auditory prediction

## Abstract

The influence of delayed auditory feedback on action evaluation and execution of real-life action-induced sounds apart from language and music is still poorly understood. Here, we examined how a temporal delay impacted the behavioral evaluation and neural representation of hurdling and tap-dancing actions in a functional magnetic resonance imaging (fMRI) experiment, postulating that effects of delay diverge between the two, as we create action-induced sounds intentionally in tap dancing, but incidentally in hurdling. Based on previous findings, we expected that conditions differ regarding the engagement of the supplementary motor area (SMA), posterior superior temporal gyrus (pSTG), and primary auditory cortex (A1). Participants were videotaped during a 9-week training of hurdling and tap dancing; in the fMRI scanner, they were presented with point-light videos of their own training videos, including the original or the slightly delayed sound, and had to evaluate how well they performed on each single trial. For the undelayed conditions, we replicated A1 attenuation and enhanced pSTG and SMA engagement for tap dancing (intentionally generated sounds) vs. hurdling (incidentally generated sounds). Delayed auditory feedback did not negatively influence behavioral rating scores in general. Blood-oxygen-level-dependent (BOLD) response transiently increased and then adapted to repeated presentation of point-light videos with delayed sound in pSTG. This region also showed a significantly stronger correlation with the SMA under delayed feedback. Notably, SMA activation increased more for delayed feedback in the tap-dancing condition, covarying with higher rating scores. Findings suggest that action evaluation is more strongly based on top–down predictions from SMA when sounds of intentional action are distorted.

## Introduction

Most human actions produce sounds, and these sounds are either the goal of the performed action (goal-related action-induced sounds, *G sounds* hereafter) or a mere byproduct (byproduct action-induced sounds, *B sounds*). This difference suggests diverging neural implementation as well, especially regarding areas that serve the selection and execution of action goals. For instance, we would expect that a tap-dancing sound (a G sound) is part of the brain’s action goal representation, whereas the sound generated by a hurdling action (a B sound) is rather not. However, when performing an action that generates a sound as a mere byproduct (a *B action*, hereafter), we would be starkly surprised if the corresponding sound would not ensue, suggesting that B sounds are part of the brain’s expectations during action execution.

Physiological evidence for this view is provided by sensory attenuation to self-initiated sounds, which manifests in electroencephalography (EEG) as amplitude reduction of the N1 component (Baess et al., 2011), and in magnetoencephalography (MEG) as amplitude reduction of the magnetic counterpart of the N1, called M100 or N1(m) component (Aliu et al., 2009; Horváth et al., 2012), which is mainly (but not exclusively, cf. Godey et al., 2001; Yvert et al., 2005) evoked from primary auditory cortex (Reite et al., 1994). Functional MRI studies indicate that this attenuation reflects decreased activity in the A1 (Straube et al., 2017). Top–down modulations causing this suppression are conceived of as predictive models, which are formed in higher cortical areas and conveyed to the respective sensory cortices to minimize prediction errors (Friston, 2005). Especially, premotor areas are associated with a forward model, which is important for the precise predictions about anticipated action outcomes, whether those are represented independently of their modality (Schubotz and von Cramon, 2003) or specifically as auditory effects (Waszak et al., 2012; Woods et al., 2014). With regard to self-produced sounds, dynamic causal modeling of event-related brain potentials in EEG suggest that A1 is modulated by predictive models from supplementary motor area (SMA) (Jo et al., 2019) and posterior superior temporal gyrus (pSTG) (Chennu et al., 2016), damping responses to expected sounds (for reviews, cf. Rauschecker, 2012; Heilbron and Chait, 2018).

By experimentally modifying action-induced sounds, the effect of auditory prediction errors on action performance has been studied mostly in language and music, where the action-induced sound is the immediate goal of the action (G sounds). Delayed auditory feedback impairs the process of speaking (Howell, 2004; Sasisekaran, 2012) and musical production (Finney, 1997; Pfordresher, 2006; Pfordresher and Beasley, 2014), although professional musicians seem to be less affected (van der Steen et al., 2014). Qualitative manipulations of action-induced sounds, e.g., manipulation of loudness or formant manipulation, evoke compensatory articulation while speaking (Bauer et al., 2006; Purcell and Munhall, 2006; Tourville et al., 2008) and singing (Jones and Keough, 2008; Keough and Jones, 2009). This interfering influence is suggested to be either caused by a distorted feedback signal in higher cortical areas or by the automatic activation of competing forward models (Pfordresher, 2006). In support of the latter suggestions, shifted pitch feedback was found to induce higher activity in anterior cingulate cortex (ACC), an area signaling conflict monitoring (Ridderinkhof et al., 2004), modulating activity in auditory cortex and SMA (Zarate and Zatorre, 2008; Zarate et al., 2010).

In contrast to language and music, other types of action-induced sounds are often a mere byproduct of our actions (B sounds), like the sound generated by placing a cup back on a table or the sound of our own footsteps on the ground. While it is not our subjective goal to produce an audible sound by these actions, we might still be irritated if the sound differs from our expectations, and hence, B sounds may also be part of the predictive model on a neuronal level. As for G sounds, delaying B sounds has an interfering effect on action performance. For instance, delaying the sound of walking interferes with our sense of agency (Menzer et al., 2010). Moreover, Kennel et al. (2015) found that delayed auditory feedback during the performance of hurdling interferes with performance, but only for the first trial. The authors suggest a dynamic and very fast adaptation of the predictive forward-loop, comparable to the adaptation to temporal asynchrony in judging audiovisual stimuli (Vroomen et al., 2004).

Under the premise that neural mechanisms involved in processing auditory feedback during performance would overlap with those involved in auditory feedback during audiovisual action observation, we presented audiovisual videos of hurdling and tap-dancing performance in a previous functional magnetic resonance imaging (fMRI) study. We found sensory attenuation to be less pronounced for B sounds compared to G sounds, and higher cortical areas, especially the SMA, were more strongly involved in the processing of G compared to B sounds (Heins et al., 2020). Thus, B actions may entail less predictive activity in the auditory system, and B sounds may be less relevant for adjusting forward models compared to G sounds. Further highlighting the importance of SMA in the predictive hierarchy of action-induced sounds, this region was found to activate sensorimotor associations regarding action-induced sounds (Lima et al., 2016).

Building on these findings and adopting the same premise, we examine the impact of delaying self-produced G and B sounds on their neural processing and the performance evaluation of G and B actions. To this end, we trained our participants in hurdling and tap dancing to establish a sensorimotor representation of these actions. In the subsequent fMRI experiment, participants were then presented with point-light videos of their own hurdling and tap-dancing performances (factor ACTION) either with their original sound or with their sound 200 ms delayed (factor DELAY), comparable to delays used in other studies (Menzer et al., 2010; Kennel et al., 2015). A number of four to six videos of the same delay type (delayed, undelayed) were presented consecutively to examine adaptation effects. Participants were instructed to rate the quality of their own hurdling and tap-dancing performance on a trial-by-trial basis.

Behaviorally, we expected the delayed presentation of sounds to lower rating scores for both B sound-generating actions (*B actions*) and G sound-generating actions (*G actions*) (Hypothesis 1). In addition, this decrease in rating scores was expected to be larger and to persist for repeated presentation of delayed G compared to delayed B sounds (Hypothesis 2).

With regard to the blood-oxygen-level-dependent (BOLD) response effects, we hypothesized that A1 should be more activated, that is, less attenuated, for the B actions with undelayed sound compared to G actions with undelayed sound, whereas regions providing the predictive forward model, namely, pSTG and SMA, should be more active for G actions compared to B actions in general (Heins et al., 2020, Hypothesis 3). Please note that we adhere to the commonly used term *posterior temporal gyrus* or *pSTG* to refer to the most posterior segment of the superior temporal gyrus, whereas we use the term *primary auditory cortex* or *A1* exclusively to refer to Heschl’s gyrus (or transverse temporal gyrus), which is of course also part of the superior temporal region but clearly buried within the lateral sulcus. We expected that the presentation of the delayed conditions would generate a prediction error, especially earlier compared to later presentations. The pSTG and also the ACC should reflect this error signal in the form of an activation increase (Hypothesis 4). Additionally, SMA activation should increase especially for the delayed G actions. Here, at the apex of the hierarchy, the predictive model is adapted to cope with the changed auditory input. This is essential when sounds are integral for action evaluation as are G sounds, whereas delayed B sounds can be ignored and should therefore lead to a less pronounced SMA response (Hypothesis 5). In both delayed conditions, activity in the SMA and pSTG should be positively correlated, as the SMA is suggested to regulate the error signal in the pSTG via top–down predictions (Hypothesis 6).

Finally, SMA activation should positively correlate with behavioral rating scores in G, but not in B actions, due to the predictive input provided by this region whenever the produced sound is an integral part of the action, guaranteeing a positive evaluation of observed actions even when the sound differs from what we expect (Hypothesis 7).

## Materials and Methods

The current fMRI study was the third in a set of three fMRI experiments pertaining to a research project in which we tested an extensively trained sample of participants and generated stimulus material that was tailored to each individual participant. Thus, stimulus set and presentation aspects overlap between the three experiments (as described in the following “Participants” section to the “Behavioral Test and Retest Sessions” section, except for the delayed stimuli in the “Material” section), whereas no data point has been used twice in any of the studies and corresponding articles.

**FIGURE 1:**
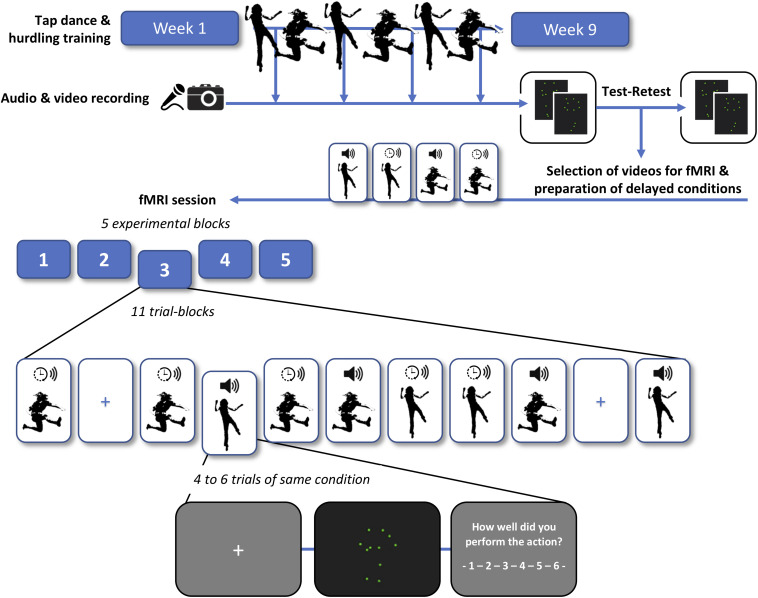
Schema of the training schedule, recording and processing of point-light videos, test–retest rating of the videos, and functional magnetic resonance imaging (fMRI) experiment. Participants were trained in tap dancing (an action in which sounds are generated intentionally) and hurdling (where sounds are generated incidentally). In a second step, each participant engaged in a test and retest rating of their own point-light videos, subjectively indicating how well they performed in each trial. We then selected the most consistently rated videos for the subsequent fMRI session. In half of the videos, sound was shifted to a time lag of 200 ms relative to the visual stimulus (indicated with a clock symbol), whereas the other half was presented with the original sound (indicated with a speaker symbol). Four experimental conditions were employed in the fMRI experiment: tap dancing with undelayed sound, tap dancing with delayed sound, hurdling with undelayed sound, and hurdling with delayed sound (see Supplementary Material for exemplary videos). To investigate the effects of auditory prediction errors and potential adaptation processes, four to six trials of the same condition were presented in trial blocks.

Figure 1 provides a schematic overview of the training schedule, recording and processing of point-light videos, test–retest of the videos, and the final fMRI experiment.

### Participants

Nineteen participants started the hurdling and tap-dancing training sessions. Eighteen of them finished the 9-week training period, and therefore, their video and audio data were processed. Four participants dropped out of the study after the training and one additional participant after the first fMRI session (Heins et al., 2020). Hence, 13 participants completed this fMRI session. One participant was excluded from the final analysis because their reaction times recorded during the fMRI session diverged more than two standard deviations from the mean reaction time on a group level, leaving 12 participants (eight females, four males) for the analysis. This is a relatively small sample size, but we found robust results in our preceding fMRI study using the same sample size (Heins et al., 2020). The participants’ age ranged from 19 to 28 years (*M* = 22.1, SD = 3.0), and all of them were right-handed, as assessed by the Edinburgh Handedness Inventory (EHI; Oldfield, 1971), with scores varying from +60 to +100, with a mean of +85. All participants reported to have no history of psychiatric or neurological disorders. They signed an informed consent. After successful participation, participants were rewarded with both course credit and monetarily. The study was approved by the local ethics committee of the University of Münster, Germany, in accordance with the Declaration of Helsinki.

### Material

The stimuli used in the study consisted of point-light videos of hurdling and tap-dancing actions with their corresponding sounds, or sound delayed by 200 ms, recorded from each individual participant at different stages throughout training (see section “Training and Filming Sessions” for further information regarding the training environment). To generate point-light videos, kinematic measurements were conducted using passive (retroreflective) markers and nine optical motion capture cameras (Qualisys opus 400 series) of the Qualisys Motion Capture System^1^ (Qualisys, Gothenburg, Sweden) (see Figure 1). At the same time, the sound was recorded by in-ear microphones (Sound-man OKM Classic II) for hurdling and by a sound recording app on a mobile phone for tap dancing. The mobile phone was handheld by a student assistant sitting about 1 m behind the tap-dancing participant. After data acquisition, point-light videos were processed using the Qualisys Track Manager software (QTM 2.14), ensuring visibility of all 14 recorded point-light markers during the entire recording time (for an overview of the position of the point-light markers, see Figure 1). Note that we excluded videos containing movement errors, e.g., touching of a hurdle. Correspondingly, all sounds in both actions were produced by foot-ground contacts only.

Sound data were processed using Reaper v5.28 (Cockos Inc., New York, NY, United States). Stimulus intensities, i.e., loudness of the stimuli, of hurdling and tap-dancing recordings were normalized separately. The spectral distributions of both recording types were then equalized by capturing the frequency profiles of hurdling and tap-dancing sounds using the Reaper plugin Ozone 5 (iZotope Inc., Cambridge, MA, United States). The plugin’s match function used the difference curve (hurdling–tap dancing) to adjust the tap-dancing spectrum to the hurdling reference. Point-light displays and sound were synchronized, and the subsequent videos were cut using Adobe Premiere Pro CC (Adobe Systems Software, San Jose, CA, United States). The final videos had a size of 640 × 400 pixels, 25 frames per second, and an audio rate of 44,100 Hz. A visual fade-in and a fade-out of 1 s (25 frames) were added with Adobe Premiere. Video length ranged from 3 to 6 s, with an average length of 5 s. Videos of tap dancing included approximately 20 tap steps, whereas hurdling videos included three hurdle clearances and approximately 16 steps.

For the fMRI session, a subset of 48 individual hurdling and 48 tap-dancing videos was selected for each participant. This subset included the 27 hurdling and 27 tap-dancing videos from our previous fMRI study (Heins et al., 2020), and 21 additional videos per action type, choosing the videos with the most reliable ratings, provided by the participants in the behavioral test–retest sessions (as described in detail in the section “Behavioral Test and Retest Sessions”).

For every selected video, a delayed sound version was created using Adobe Premiere. The sound was adjusted to start with a 200-ms (five frames) delay in reference to the beginning of the video. All videos were presented using the Presentation software (Version 18.1, Neurobehavioral Systems, Inc., Berkeley, CA, United States).

### Procedure

#### Training and Filming Sessions

Participants were trained in hurdling and tap dancing by professional instructors for a 9-week period. The training took place at the movement laboratory OpenLab of the Institute of Sport and Exercise Sciences at the University of Münster, located inside a ball sports hall. Participants trained both action types each for 3 h a week, participating in two 90-min training sessions a week for hurdling and in two 90-min training sessions a week for tap dancing. None of the participants had any experience in hurdling and tap dancing before starting the training. There was one instructor for the training of tap dancing and one out of four instructors for each training session of hurdling. Both tap-dancing and hurdling training were conducted in small groups. The tap-dance sequence consisted of standard taps that are usually selected to build up a beginner’s repertoire. Hurdling included three hurdle clearances, with a training-level-dependent spatial distance between hurdles (this was about 8 m at the final level of training).

Participants had to take part in four out of six offered filming sessions. The first filming sessions took place 2 weeks after the training started, with the following sessions taking place 4, 5, 6, 8, and 9 weeks after training starts. During these sessions, participants were equipped with 14 point-light markers (see Figure 1), which were tracked via infra-red cameras of the motion-capturing system while performing both action types. No motion capture markers were used for the hurdles. The hurdling action consisted of three hurdle clearances (Figure 1), while the tap-dancing action was a movement sequence learned during the tap-dancing training sessions. Both actions increased in difficulty within the four sessions. For hurdling, the spatial distance between the three hurdles increased, requiring more speed and a smoother hurdle clearance. For tap dancing, action elements were added to the sequence to increase difficulty.

#### Behavioral Test and Retest Sessions

Behavioral test–retest sessions were conducted to determine the stimuli with the highest reliability of participants’ rating. Rating was conducted by the same participants who already engaged in the training (cf. the sections “Material” and “Training and Filming Sessions”), and every participant rated only point-light videos generated from their own movements during the training sessions. Test–retest sessions were conducted in a computer lab. Participants were seated in front of a computer and instructed to rate the quality of their actions on a scale from 1 (“*not well at all*”) to 6 (“*very well*”) based on their subjective impression. We did not bias participants’ judgments in view of the visual or the auditory domain; instead, we simply asked them to indicate after each video how well they think they performed in this video. The experiment consisted of two blocks with self-paced responses, with both blocks lasting between 20 and 30 min. The same videos were presented in a different order in the two blocks of the experiment. Videos were pseudorandomized so that not more than three videos in a row showed the same action type (hurdling vs. tap dancing). Overall durations of all test sessions ranged from 40 to 60 min, depending on rating speed. Two weeks after the first test session, participants were presented the same videos again (in pseudo-randomized order). Forty-eight videos for both hurdling and tap dancing were chosen per participant and were used in the current fMRI session. The videos with the highest reliability in ratings were chosen. Each video was rated a total of four times (two times in the test and two times in the retest sessions). Of all chosen videos (672 videos in total, 96 per participant), 16.52% received the same rating on all four repetitions. In 61.76% of the chosen videos, ratings varied by a score of either +1 or −1 in one or two of the repetitions (ratings diverging in one direction), and in 21.72% of the chosen videos, ratings varied by a score of ±1 (ratings diverging in both directions). Note that, as explained above, 27 hurdling videos and 27 tap dancing videos were used in a preceding fMRI study (Heins et al., 2020).

#### Functional Magnetic Resonance Imaging Session

For the fMRI session, participants completed the same task as during the test–retest sessions, namely, to rate the quality of the presented actions on a six-point Likert scale. Since we did not intend to draw the participants’ attention to the delayed sounds, they were not informed about the presence of videos with delayed sound. Participants were asked to regulate the volume of the sounds before the experiment started to assure that the action-induced sounds were audible above the scanning noises.

The experiment consisted of five blocks, including 11 trial blocks each. Trial blocks consisted of four to six trials of the same condition. Transition probabilities ensured that each condition was preceded by every condition (including the same condition) in the same number of trial blocks over the whole experiment. The first trial block of a block was a repetition of the last trial block of the preceding block, to avoid losing a transition. The remaining 10 trial blocks consisted of two trial blocks for each of the five conditions (namely, hurdling undelayed, tap dancing undelayed, hurdling delayed, tap dancing delayed, and null events). Thus, after the discarding of the first trial block after each pause, 50 trial blocks, containing 240 trials, remained for the analysis. These consisted of 192 video trials (48 trials per conditions) and 48 null events, where a fixation cross was presented. The duration of the null events was 5 s. Before each trial, a fixation cross was presented as an interstimulus interval, varying between 3.5 to 4.5 s in length. After each video trial, the rating scale from 1 to 6, including the rating question, was presented. The experiment continued upon the participants’ button press.

Throughout the entire scanning routine, participants were instructed to refrain from moving.

### Functional Magnetic Resonance Imaging Recordings and Preprocessing

Participants were scanned in a 3-Tesla Siemens Magnetom Prisma MR tomograph (Siemens, Erlangen, Germany) using a 20-channel head coil. A 3D-multiplanar rapidly acquired gradient-echo (MPRAGE) sequence was used to obtain high-resolution T1-weighted images ahead of functional scanning, with scanning parameters set to 192 slices, a repetition time (TR) of 2,130 ms, an echo time (TE) of 2.28 ms, a slice thickness of 1 mm, a field of view (FoV) of 256 mm × 256 mm, and a flip angle of 8°.

Gradient-echo echoplanar imaging (EPI) was used to measure BOLD contrast for functional imaging data of the whole brain. There were six EPI sequences in total; one sequence for the volume adjustment and one sequence for each of the five experimental blocks. Scanning parameters were set to a TE of 30 ms, a TR of 2,000 ms, a flip angle of 90°, 33 slices were acquired interleaved with a slice thickness of 3 mm, and an FoV of 192 mm × 192 mm.

Imaging data were processed using SPM12 (Wellcome Trust, London, United Kingdom). Slice time correction to the middle slice was performed, followed by realignment of all individual functional MR (EPI) images to correct for t3D motion. The individual’s structural scan was coregistered to the mean functional image and then segmented into the native space tissue components. Both the structural and the functional images were normalized into the standard MNI space (Montreal Neurological Institute, Montreal, QC, Canada). Spatial smoothing of the functional images was performed with a Gaussian kernel of full-width at half maximum (FWHM) of 8 mm. To additionally reduce effects of motion, we performed a denoising procedure on the EPI data using the default settings of the CONN toolbox in MATLAB (Whitfield-Gabrieli and Nieto-Castanon, 2012), which implements the anatomical component-based noise correction method (aCompCor). Denoising included regressing out the first five principal components associated with white matter and cerebrospinal fluid as well as the motion parameters and their temporal derivatives from the BOLD signal. A high-pass temporal filter equivalent to 128 s was applied to the data.

### Statistical Data Analysis

#### Behavioral Data Analysis

We calculated Kolmogorov–Smirnov tests to assure the normal distribution of our behavioral rating score data. To examine a potential reduction in rating scores by initial sound delay in general (Hypothesis 1), we calculated 2 × 2 × 3 repeated measures analyses of variance (rmANOVA) on mean rating scores using SPSS (IBM, New York, NY, United States). To examine adaptational effects, which are reflected in restored rating scores and happen very fast (cf. Kennel et al., 2015, for performance restoration after the first delayed trial), we only included the first three trials of each trial block in the analysis, so that the rmANOVA included the factor ACTION, with the factor levels *B* (*hurdling*) and *G* (*tap dancing*), the factor DELAY with factor levels *undelayed* and *delayed*, and the factor REPETITION with factor levels *first*, *second*, and *third presentation*, referring to the first three consecutively presented trials of each trial block. We expected a significant main effect for DELAY (Hypothesis 1). Testing for the diverging impact of sound delay on B and G actions, i.e., the stronger decrease in rating scores for delayed G sounds and the persisting effect of this delay over several consecutive trials (Hypothesis 2), we additionally hypothesized an interaction effect ACTION × DELAY, and an interaction effect ACTION × DELAY × REPETITION.

To further examine significant interaction effects, factors were held constant, and simple main effects as well as paired *t*-test were calculated. The significance level was set to α = 0.05 and Bonferroni correction was performed for multiple comparisons.

#### Functional Magnetic Resonance Imaging Design Specification

The design was implemented in SPM12, following a general linear model (GLM, Friston et al., 1994; Worsley and Friston, 1995) approach. The modeled activation was time-locked to the onsets of the videos for each of the experimental conditions or the null events. Epochs contained the full presentation period ranging from 3 to 6 s for the videos, and 5 s for the null events. For the delayed conditions, we added the number of repetitions within a trial-block, indicating the first, second, third, … up to sixth delayed sound video presented in a row, as parametric modulators to the respective regressor in order to examine the initial interference and adaptational processes. The GLM for every participant thus consisted of 16 regressors in total: four regressors for the experimental conditions (B undelayed, G undelayed, B delayed, G delayed), two parametric regressors modeling the number of repetitions for the two delayed conditions (repetition B delayed, repetition G delayed), one regressor for the null events, and six regressors for the motion parameters (three translations and three rotations). Activation for 48 trials was considered for the modeling of each of the four regressors for the experimental conditions and for the null event regressor. All these regressors were convolved with the hemodynamic response function.

On the first level, *t*-contrasts of the experimental conditions against null events were calculated (condition > rest). These contrast images were then used to set up a flexible factorial design on the second level. The flexible factorial design was chosen because it accounts best for the within-subject factors. The model consisted of 16 regressors—four regressors for the experimental conditions and 12 regressors for the subject effects, one for each participant. Additionally, *t*-contrasts for the parametric regressors were calculated on the first level and one-sample *t*-tests on the second level. Subsequently, we corrected for multiple comparisons using the false discovery rate (FDR) method controlling the expected proportion of false positives among suprathreshold voxels at *p* < 0.05.

Region of interest (ROI) analyses were performed using the WFU PickAtlas toolbox (Maldjian et al., 2003) in SPM12. On this basis, we tested our hypotheses and sought to replicate results from our previous fMRI study (Heins et al., 2020) from which we derived functional ROIs using peak voxels of the right primary auditory cortex (*x* = 54, *y* = −13, *z* = 5), right pSTG (*x* = 54, *y* = −31, *z* = 5), and SMA (*x* = −3, *y* = −4, *z* = 68). ROIs were defined as spheres of 6 mm around these peak coordinates. To test for the hypothesized stronger sensory attenuation for tap dancing (G action) than for hurdling (B action), we performed an ROI analysis focusing on the primary auditory cortex for the B > G contrast including only trials with undelayed sound as sensory attenuation should only arise when sounds are undelayed and thus predictable. Then, ROI analyses for the secondary auditory cortex (pSTG) and the SMA for the G > B contrasts were performed, including both delayed and undelayed sound conditions to test a stronger top–down predictive signal for tap dancing than for hurdling (Hypothesis 3).

To test for effects of the prediction error elicited in ACC and pSTG by delayed sound videos (Hypothesis 4), we first calculated the contrast delayed > undelayed. Considering the possibility that delayed sound does not evoke a persisting prediction error, but that the error signal is strongest for the first presentation of delayed sound (cf. Kennel et al., 2015), we additionally calculated a one-sample *t*-test for the effects of parametric regressors (repetition B delayed, repetition G delayed) on the second level. This regressor was suggested to capture the transient increase and subsequent decline of activation caused by the first delayed sound trial within a trial-block.

To test Hypothesis 5, we extracted beta values from the SMA to test for the adaptational shift to the predictive model evoked by the delayed sound videos, especially in the delayed G condition, and examined the expected correlation between pSTG and SMA beta values for both delayed sound conditions (Hypothesis 6). Finally, assuming that SMA provides predictive input to ensure proper performance evaluation even when sound is delayed, we tested for a positive correlation between SMA beta values and rating scores for G sounds but not B sounds (Hypothesis 7). Significance tests of the correlational analyses were performed at α = 0.05, one-sided, based on directional hypotheses.

## Results

### Behavioral Results of the Functional Magnetic Resonance Imaging Session

A 2 × 2 × 3 rmANOVA with the factors ACTION (*B, G*), DELAY (*undelayed*, *delayed*), and REPETITION (*first, second, third presentation in trial-block*) yielded no main effect for DELAY [*F*(1,11) = 3.77, *p* = 0.078]. Contrary to Hypothesis 1, rating scores increased slightly (but only descriptively) for the delayed conditions (*M* = 3.59, SD = 0.66) compared to the undelayed sound conditions (*M* = 3.45, SD = 0.59). Neither the interaction ACTION × DELAY [*F*(1,11) = 0.04, *p* = 0.847] nor the interaction ACTION × DELAY × REPETITION [*F*(1,11) = 1.22, *p* = 0.335] were significant. Descriptively, ratings decreased for the delayed B actions from the first (*M* = 3.78, SD = 0.58) to the second (*M* = 3.67, SD = 0.61) to the third presentation (*M* = 3.52, SD = 0.66), while the opposite was observed for the delayed G actions (first presentation *M* = 3.41, SD = 1.02; second presentation *M* = 3.51, SD = 0.95; third presentation *M* = 3.65, SD = 1.03).

### Functional Magnetic Resonance Imaging Results

**FIGURE 2:**
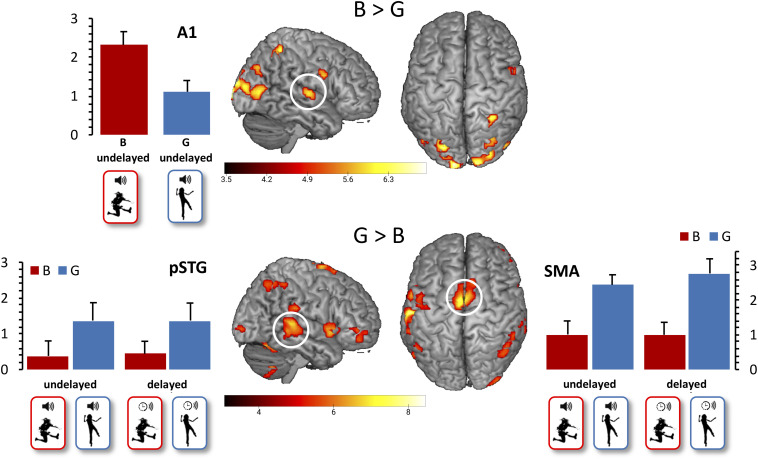
Main effect of action. False discovery rate (FDR)-corrected t-maps (*p* < 0.05) for the B > G contrast are presented in the top row. FDR-corrected t-maps (*p* < 0.05) for the G > B contrast are presented in the bottom row. Additional beta values for the primary auditory cortex, the pSTG, and the SMA are presented, with mean beta values for the B conditions presented in red, and mean beta values for the G conditions presented in blue. Error bars indicate standard errors of the mean.

With regard to our first functional hypothesis (Hypothesis 3), the whole-brain contrast G > B revealed activation in the SMA (*x* = 0, *y* = −4, *z* = 68) and pSTG (*x* = 48, *y* = −34, *z* = 5), as expected. The expected stronger A1 activation for B vs. G action-induced sounds was not revealed when restricted to undelayed action-induced sounds, but became significant (*x* = 51, *y* = −13, *z* = 5) when contrasting all B vs. G actions after FDR correction to control the expected proportion of false positives among suprathreshold voxels at *p* < 0.05 (Figure 2). Moreover, beta values extracted from A1 differed significantly between the B (*M* = 2.32, SD = 1.18) and the G conditions (*M* = 1.11, SD = 1.01, *t*(11) = 2.79, *p* = 0.018), determined by a paired sample *t*-test between B and G with undelayed sound.

**FIGURE 3:**
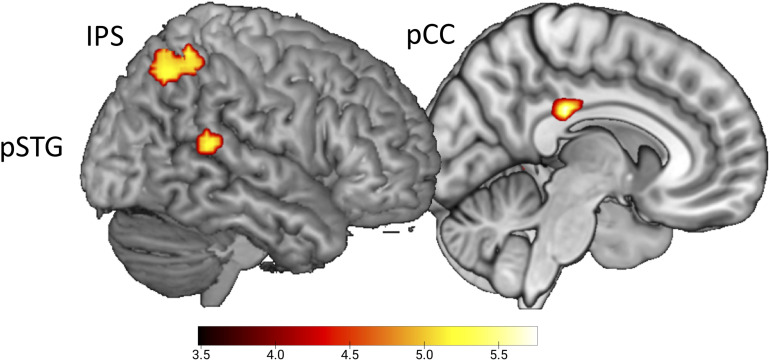
Initial effect of delayed sound. Uncorrected t-maps (*p* < 0.001) for the parametric effect of repetition for the delayed conditions. Activations are evoked by the initial vs. repeated presentation of videos with delayed sound (both for B and G actions).

To test for the expected error response by the presentation of conditions with delayed sounds (Hypothesis 4), the whole-brain contrast delayed > undelayed, did not reveal significant effects. In contrast, the conjoined effect of the parametric regressors for delayed B and G sounds, emphasizing the response to the first delayed sound video in a row, revealed activation in pSTG (*x* = 54, *y* = −43, *z* = 14), the intraparietal sulcus (*x* = 36, *y* = −52, *z* = 53), and the posterior cingulate cortex (pCC, *x* = 9, *y* = −28, *z* = 29, uncorrected, *p* < 0.001, Figure 3).

Beta values extracted from SMA showed a trend for the interaction effect ACTION × SOUND [*F*(1,11) = 3.78, *p* = 0.078]. Descriptively, the adaptational effect in SMA was slightly stronger for G than for B actions, as expected (Hypothesis 5), although *post hoc t*-tests were only approaching significance (*p* > 0.10).

By testing for a positive correlation of beta scores in SMA and pSTG for the delayed G and B conditions (Hypothesis 6), we found a non-significant trending correlation for B sounds (*r* = 0.467, *p* = 0.063) and for G sounds (*r* = 0.390, *p* = 0.105), but no correlations for undelayed B sounds (*r* = 0.221, *p* = 0.245) or undelayed G sounds (*r* = 0.164, *p* = 0.306). Finally, we calculated correlations between the SMA beta values and rating scores for the delayed B and G conditions (Hypothesis 7). Here, we did not find a significant correlation for delayed B sounds (*r* = −0.363, *p* = 0.123) as expected, but a non-significant trend for delayed G sounds (*r* = 0.412, *p* = 0.092).

## Discussion

It strikes us when a sound differs from what we expected—especially when it is generated by our own actions. Starting from the assumption that this surprise is larger when we actively intend to create a sound, compared to when the sound is an incidental byproduct of our performed action, we here used fMRI to examine how the performance rating and the neuronal processing of one’s own action videos are affected when the sound playback is delayed. Videos were recorded from our participants during a 9-week training schedule of two whole-body actions–hurdling, operationalizing incidental action-induced sounds (B sounds), and tap dancing, operationalizing intentional action-induced sounds (G sounds). We found a statistically moderate error response to the delayed playback of both types of action-induced sounds in pSTG, the intraparietal sulcus, and pCC, and SMA showed a descriptive (although non-significant) trend to being more engaged for delayed G sounds and their behavioral performance evaluation, as discussed below.

Comparing rating scores for actions presented with correctly timed or 200 ms delayed sound, we had two hypotheses: first, that rating scores would decrease, i.e., action performance is perceived as worse, for the delayed sound conditions (Hypothesis 1) and second, that this decrease would be especially prominent and persistent for G actions, as action-induced sounds were expected to be more important for the rating of G actions (Hypothesis 2). Neither of these hypotheses were corroborated by the data. Descriptively, rating scores for the delayed sound conditions even tended to be higher than for the undelayed sound conditions. Studies examining audiovisual asynchrony showed that a small audio-lag results in higher perceived synchrony ratings than synchronous presentation (Vatakis and Spence, 2006; Eg and Behne, 2015). Thus, the delayed conditions were possibly perceived as more synchronous and therefore rated slightly more positive. The employment of a delayed sound condition was not mentioned during participants’ instruction, and when asked if they noticed anything in a post-fMRI survey, only 2 of the 12 participants reported to have perceived a delay in some of the videos. Together with the non-significant behavioral effects, this could suggest that the delay of 200 ms we selected was actually too short to yield more robust effects. This is different from previous findings. A 180-ms delay in action-induced sounds caused at least a temporally worse performance in hurdling (Kennel et al., 2015), and delayed auditory feedback longer than 120 ms significantly decreased the sense of agency during walking (Menzer et al., 2010). It is possible that delayed auditory feedback has a less pronounced effect when presented offline in a video presentation setting rather than during real performance, as in these two studies. In favor of this assumption, kinematic familiarity of a dance movement was observed to increase the perceived coherence with a corresponding auditory beat, making a slight auditory shift in time less noticeable (Su, 2018). Generalizing this observation further, strong expectations of coherence appear to hinder the detection of audiovisual asynchrony (Su, 2014). Thus, the consistent motor expertise in our sample might have led to a positive bias for audiovisual integration, leading to unaffected rating scores for even the delayed sound conditions.

This said, the selected delay of 200 ms was obviously not too short to generate an increased brain response to the delayed sound conditions, as discussed in the following, suggesting that performance evaluation was more challenging in the absence of actions sounds. Crucially, an increased BOLD response to more challenging task conditions do not necessarily involve behavioral decline. Thus, the combination of preserved behavioral performance and increased BOLD responses is a hallmark of successful compensatory processes, as for instance reported in elderly (Cabeza, 2001) or brain-damaged patients (Stern, 2017).

As to the results of brain activation, a caveat for the present discussion is that the sample size was small due to a relative high dropout rate. This dropout could not be easily compensated since participants enrolled in a complex, 9-week-long training including several video recordings, and a time-consuming preparation of the stimulus material tailored to each individual participant. Except for one unexpected finding in the parametric effect of repetition, we confine our discussion to only hypothesized areas. In view of slightly underpowered data, it was particularly important to find that we could replicate a previous study (Heins et al., 2020) observing stronger activation of SMA and pSTG in the tap-dancing condition and higher activation—i.e., less attenuation—in primary auditory cortex in the hurdling condition (Hypothesis 3). We take this result to strengthen the assumption that the brain does not engage in prediction of B sounds as much as G sounds. This interpretation is also supported by the observation that SMA and pSTG provide stronger top–down modulations of primary auditory cortex in the case of action-induced sounds produced by voluntary actions (Reznik et al., 2018). An alternative explanation is that B sounds may be simply less *predictable* (not less predicted *per se*) than G sounds, as a sound’s predictability has been found to co-vary with attenuation in primary auditory cortex (Straube et al., 2017). However, we take this alternative account to be less convincing as our participants had intensively trained for both B and G actions; they had rated all of the videos two times in the test–retest sessions and therefore could be considered being quite familiar with both types of stimuli.

Since posterior temporal areas have been suggested to play a role for audiovisual integration, it is important to consider our findings in light of this finding as well. One may speculate that the processing of videos presenting incidentally and intentionally produced sounds may differ with regard to the demands they pose on audiovisual integration. Indeed, our own behavioral pilot studies suggest a bias toward synchronous vs. asynchronous judgments for tap dancing compared to hurdling. Marchant et al. (2012) found both A1 (*x* = −54, *y* = −21, *z* = 6) and pSTG (*x* = −63, *y* = −45, *z* = 6) to increase for synchronous vs. asynchronous audiovisual stimulus trains, including a pSTG ROI (*x* = −54, *y* = −50, *z* = 8) taken from a former study (Noesselt et al., 2007); authors could also demonstrate that synchrony, not predictability, gave rise to this area’s engagement in audiovisual integration. In clear contrast to this coactivation, our experimental conditions disentangled A1 (*x* = 51, *y* = −13, *z* = 5) and pSTG (*x* = 48, *y* = −34, *z* = 5) modulation, leading to higher A1 engagement in hurdling vs. tap dancing (i.e., B vs. G actions) but, at the same time, to higher pSTG engagement in tap dancing vs. hurdling (i.e., G vs. B actions). Consequently, higher perceived synchronicity can hardly explain differences between tap dancing and hurdling in our study. Even when leaving coactivation of A1 aside, a directly adjacent field in pSTS (*x* = 54, *y* = −43, *z* = 14) increased for the delayed sound conditions, as hypothesized and discussed in the following, which bluntly rules out an explanation along the lines of higher perceived synchronicity.

As expected, we found pSTG to provide a significant error signal to the first presentations of the delayed sound conditions that adapted over the course of repeated presentations in the trial blocks (Hypothesis 4). This is in line with studies regarding pSTG as an auditory error detector responding to altered feedback (Fu et al., 2006; Zheng et al., 2010; Johnson et al., 2019). In one of our previous behavioral studies, we presented delayed auditory feedback during the actual performance of hurdling (Kennel et al., 2015) and found an interfering effect on the first hurdling trial, whereas performance restored to normal in subsequent trials. With the caveat of indirect inference, this pattern resembles the subtle error response to delayed sound in the present fMRI study that declined when presenting further three to five delayed sound trials.

While we had expected the same response pattern in ACC, reflecting an error signal in action monitoring (Van Veen and Carter, 2002), it was actually present in the pCC. Since pCC was not hypothesized—in contrast to the pSTG effect—and the parametric contrast testing Hypothesis 4 was reported at *p* < 0.001 (uncorrected), discussing the pCC effect is speculative and remains to be corroborated by future studies. Whereas the entire cingulate cortex is engaged in the control of action, relating emotion, action, and memory, the posterior and anterior cingulate differ regarding their specific contribution. In contrast to the ACC, which is more related to limbic aspects such as reward and action outcome, pCC seems more related to memory and learning (Rolls, 2019). The pCC receives visuospatial and somatosensory action-related information from the dorsal stream, including parietal cortex and hippocampus, that probably serve action–outcome learning (Rolls, 2019). Moreover, pCC is also engaged in tasks that require self-imagery (Johnson et al., 2002; Kircher et al., 2000, 2002; Sugiura et al., 2005) and self-referential processing (Northoff et al., 2006). Interestingly, pCC was found to be particularly engaged when self-generated sensory consequences are temporally deviant (cf. Straube et al., 2017). Against this backdrop, we speculate that pCC provides a specific error response to the delayed conditions in our data, reflecting deviation from the learned auditory effects of participants’ hurdling and tap dancing.

We found SMA beta values to be specifically higher for the delayed G sounds, according to Hypothesis 5. SMA hence seems not immediately involved in detecting altered feedback, in contrast to pSTG and pCC, but rather provides additional top–down information about the intended action outcome whenever perceived outcome differs from the expected. Being critical for audio–motor associations (Lima et al., 2016; Johnson et al., 2019), the SMA might get engaged when auditory delays persist over a longer time to modify audio–motor models or to amplify other modalities for motor control. Accordingly, pSTG and SMA beta values also tended to be correlated in the delayed conditions, but not in the undelayed conditions (Hypothesis 6). Along the lines of a predictive coding account, this effect may be taken to reflect an error signal caused by delayed G sounds conveyed from the pSTG to the SMA to adjust higher-order audio–motor models (cf. Heilbron and Chait, 2018). In the present study, this process only manifested in case of G sounds, where we observed a non-significant trend in rating scores positively correlating with SMA beta values in the delayed condition, whereas no such correlation was found for delayed B sounds (Hypothesis 7). The SMA thus may intervene especially when the intended action-induced sounds are distorted, and accurate action-induced sound information must be restored from the model to perform the action evaluation task reliably.

Taken together, our study emphasizes the role of SMA in audio–motor processing. SMA seemingly is the apex of the action-induced sound prediction hierarchy, intervening to resolve surprise elicited by altered auditory feedback that pSTG on a hierarchical midlevel cannot resolve. This seems to be especially true for intentionally generated action-induced sounds, which are particularly important for a proper evaluation of sound-generating actions.

Our experimental design and contrasts are geared to detect potential differences between the cerebral processing of incidentally and intentionally self-produced sounds. An inevitable limitation for testing reafferent feedback in the MRI scanner is that one can, at the best, generate a stimulus and task that come as close as possible to the real movement situation. Even when our participants were viewing and hearing their own motion videos after extensive training, they performed an audiovisual task inside the MRI scanner rather than receiving delayed auditory feedback during the actual performance. Bridging this methodological gap remains a fundamental objective on the way to understanding feedback functions of auditory re-afferences.

## Conclusion

Sounds created by our own actions, though omnipresent in our everyday life, have been scarcely examined in an ecologically valid fashion. By providing participants with delayed auditory feedback when watching their own tap dancing and hurdling performance, we found behavioral and fMRI evidence for the intuitive difference between action-induced sounds, which are the intended goal of our actions and action-induced sounds created as a byproduct of performed actions. In contrast to the latter, brain responses revealed increased predictive engagement for evaluating actions with intended action-induced sounds to cope with disrupted auditory feedback. Future research may focus on a better understanding of effects of delayed auditory feedback by systematically testing when and how time matters in brain function and behavior creating a pathway for unsolved problems such as schizophrenia, stuttering, or imitating others—it is never too late.

## Data Availability Statement

The raw data supporting the conclusions of this article will be made available by the authors, without undue reservation.

## Ethics Statement

The studies involving human participants were reviewed and approved by the Ethics Committee of the Department of Psychology, University of Münster. The patients/participants provided their written informed consent to participate in this study.

## Author Contributions

NH and RS contributed to conception and design of the study. NH performed the statistical analysis and wrote the first draft of the manuscript. IT provided additional and substantial help with the data analysis. KZ, MR, and RS contributed with scientific support, supervision, and coordination. All authors contributed to manuscript revision, read, and approved the submitted version.

## Conflict of Interest

The authors declare that the research was conducted in the absence of any commercial or financial relationships that could be construed as a potential conflict of interest.

## Footnotes

1 https://www.qualisys.com
